# The relationship between watermelon consumption and sarcopenia in an elderly general population: findings from the Tianjin chronic low-grade systemic inflammation and health study

**DOI:** 10.3389/fnut.2025.1663996

**Published:** 2025-09-30

**Authors:** Xuena Wang, Yang Yang, Lin Yin, Yufei Fang, Qi Mei, Kaijun Niu

**Affiliations:** ^1^Shanxi Bethune Hospital, Shanxi Academy of Medical Sciences, Third Hospital of Shanxi Medical University, Tongji Shanxi Hospital, Taiyuan, China; ^2^Department of Ophthalmology, Renmin Hospital of Wuhan University, Wuhan, Hubei, China; ^3^Ningbo No. 2 Hospital, Ningbo, China; ^4^Department of Oncology, Tongji Hospital, Tongji Medical College, Huazhong University of Science and Technology, Wuhan, Hubei, China; ^5^School of Public Health of Tianjin University of Traditional Chinese Medicine, Tianjin, China; ^6^Nutritional Epidemiology Institute and School of Public Health, Tianjin Medical University, Tianjin, China; ^7^School of Integrative Medicine, Tianjin University of Traditional Chinese Medicine, Tianjin, China; ^8^Tianjin Key Laboratory of Environment, Nutrition and Public Health, Tianjin, China; ^9^Center for International Collaborative Research on Environment, Nutrition and Public Health, Tianjin, China; ^10^National Demonstration Center for Experimental Preventive Medicine Education, Tianjin Medical University, Tianjin, China

**Keywords:** Citrullus, sarcopenia, aged, citrulline, muscle, skeletal

## Abstract

**Background:**

Sarcopenia, a progressive skeletal muscle disorder characterized by accelerated loss of muscle mass, strength, and function, affects 10–16% of elderly individuals globally, posing a significant public health challenge. Nutrition is crucial in mitigating sarcopenia progression, with research increasingly focusing on whole foods rather than isolated nutrients. Watermelon emerges as a potentially beneficial functional food due to its high content of L-citrulline, which may support muscle health through various biological mechanisms.

**Methods:**

This population-based cross-sectional study was conducted in Tianjin, China, and analyzed 3,733 elderly participants. Sarcopenia was diagnosed using Asian Working Group for Sarcopenia criteria. Watermelon consumption was assessed using a validated semi-quantitative food frequency questionnaire, categorizing participants into three groups: “almost never,” “≤1 time/week,” and “≥2–3 times/week.” Multiple logistic regression models adjusted for demographic factors, lifestyle variables, medical history, and dietary patterns.

**Results:**

Sarcopenia prevalence was 12.6% among participants (median age: 65.8 years). Compared to non-consumers, participants consuming watermelon ≤1 time/week had 28% lower odds of sarcopenia (odds ratio [OR] = 0.72; 95% confidence interval [CI]: 0.54–0.95), while those consuming ≥2–3 times/week had 51% lower odds (OR = 0.49; 95% CI: 0.29–0.79), showing a significant inverse dose–response relationship (*p* < 0.001).

**Conclusion:**

Higher watermelon consumption was associated with lower sarcopenia prevalence in elderly Chinese adults. As this was a cross-sectional study, the findings indicate associations rather than causality, and reverse causation cannot be ruled out. Bioactive compounds in watermelon, such as L-citrulline, may be associated with muscle protein synthesis, meriting confirmation in future longitudinal and interventional studies.

## 1 Introduction

Sarcopenia has emerged as a critical geriatric syndrome with profound implications for morbidity, disability, poor quality of life, and even mortality in aging populations (1, 2). The concept of sarcopenia was first introduced by Rosenberg in the late 1990s, emphasizing the age-related loss of muscle mass as a distinct geriatric concern (3). Subsequent landmark epidemiologic research by Baumgartner et al. provided the first operational definition and prevalence estimates using appendicular skeletal muscle mass (ASM) adjusted for height, establishing sarcopenia as a measurable public health issue (4). The European Working Group on Sarcopenia in Older People (EWGSOP) has refined its diagnostic criteria over the past decade, emphasizing low muscle strength as a primary indicator, with low muscle quantity/quality and impaired physical performance further confirming the diagnosis (5). Globally, it affects approximately 10–16% of older adults (6). In China, the prevalence ranges from 14 to 20% among older adults (7–10). With global populations aging rapidly, early identification of modifiable factors, particularly nutritional interventions, is imperative.

Accumulating evidence highlights nutrition as a cornerstone in mitigating sarcopenia progression (11, 12). Nutritional intervention studies demonstrated that high protein oral nutritional supplements might be effective for certain sarcopenia-related outcomes (13, 14), prompting the integration of increasing protein intake into sarcopenia management guidelines (15, 16). Besides, essential amino acids (e.g., leucine), and micronutrients (e.g., vitamin D, antioxidants) are critical for maintaining muscle protein synthesis and reducing oxidative stress, a key driver of muscle atrophy (11, 17–21). However, recent research has shifted toward evaluating whole foods and dietary patterns rather than isolated nutrients, recognizing synergistic effects of bioactive compounds in natural food matrices (22).

Watermelon (*Citrullus lanatus*), a widely consumed fruit globally, is uniquely positioned as a functional food for sarcopenia prevention due to its high content of L-citrulline, lycopene, *β*-carotene, and vitamins A and C (23, 24). L-citrulline, a non-essential amino acid, serves as a precursor to L-arginine, enhancing nitric oxide (NO) synthesis, a vasodilator that improves blood flow to skeletal muscles and potentiates nutrient delivery (25). Moreover, watermelon’s antioxidant profile is notable. Lycopene, a potent carotenoid, mitigates oxidative stress by scavenging free radicals, thereby preserving mitochondrial function in muscle cells (26). *β*-carotene and vitamin C further synergize to reduce inflammatory markers such as interleukin-6 (IL-6) and C-reactive protein (CRP), which are implicated in muscle wasting (27). Intervention and observational studies support these mechanistic pathways in human populations. Randomized controlled trials (RCTs) have shown that L-citrulline supplementation improves muscle protein synthesis and increases lean mass in older adults, enhancing exercise performance and recovery (28, 29); F (30). Watermelon juice consumption has been reported to reduce post-exercise muscle soreness (31). In parallel, the Framingham Offspring Study demonstrated that higher carotenoid intake, including lycopene, was associated with increase in handgrip strength (HGS) and faster gait speed in this cohort of adults (27). Meta-analytic evidence confirms that diets rich in antioxidant-containing fruits are linked to about 40% lower sarcopenia risk in older adults (22). Previous evidence indicates that other fruits rich in L-citrulline and lycopene may also contribute to muscle health in older adults. Higher tomato and tomato product consumption has been associated with reduced decline in HGS and may improve muscle fiber composition and mitochondrial function through pathways such as Adenosine 5′-monophosphate activated protein kinase (AMPK) and Sirtuin 1 activation (26, 32, 33). Similarly, citrus flavonoids like tangeretin and naringin have been shown to enhance skeletal muscle mitochondrial biogenesis and promote oxidative muscle fiber remodeling via AMPK-Peroxisome proliferator-activated receptor-*γ* coactivator-1α (PGC-1α) signaling (34–36). These findings suggest that fruits containing these bioactive compounds might play a protective role against sarcopenia, providing a broader nutritional context for examining the potential benefits of watermelon, which is particularly abundant in both L-citrulline and lycopene.

Despite the biological plausibility and existing preclinical evidence suggesting the beneficial effects of watermelon or its constituents on muscle health, epidemiological data exploring the relationship between watermelon consumption and sarcopenia are scarce. Most existing studies have either focused on specific nutrients or broadly categorized fruits and vegetables without examining watermelon consumption explicitly (11, 13, 14, 17–22). Sarcopenia poses a particularly urgent challenge in China, where rapid demographic aging is occurring alongside significant shifts in dietary patterns (1). By 2040, China is projected to have over 400 million people aged 60 years and older, the largest elderly population in the world (37). Traditional diets are transitioning toward more varied but often lower fruit intake, potentially affecting sarcopenia-related risk factors (38). Understanding diet-health relationships in this context is crucial for developing culturally tailored prevention strategies. Therefore, the present study aims to explore the relationship between watermelon consumption and sarcopenia among elderly participants in the general population of Tianjin, China. We hypothesized that higher watermelon consumption would be inversely associated with sarcopenia prevalence in elderly adults, based on the biological mechanisms of L-citrulline in supporting muscle protein synthesis and the antioxidant properties of watermelon’s bioactive compounds in preserving muscle health.

## 2 Methods

### 2.1 Study design and participants

This investigation employed a population-based cross-sectional design, drawing participants exclusively from the Tianjin Chronic Low-grade Systemic Inflammation and Health (TCLSIH) Cohort Study. The TCLSIH Cohort represents a comprehensive, prospective, and dynamic cohort established to examine the associations between chronic low-grade systemic inflammation and diverse health outcomes. Detailed methodological aspects of the TCLSIH Cohort have been documented in previous publications (39). The Institutional Review Board of Tianjin Medical University granted ethical approval for this research (reference number: TMUhMEC 201430), and all participants provided written informed consent prior to enrollment.

**Figure 1:**
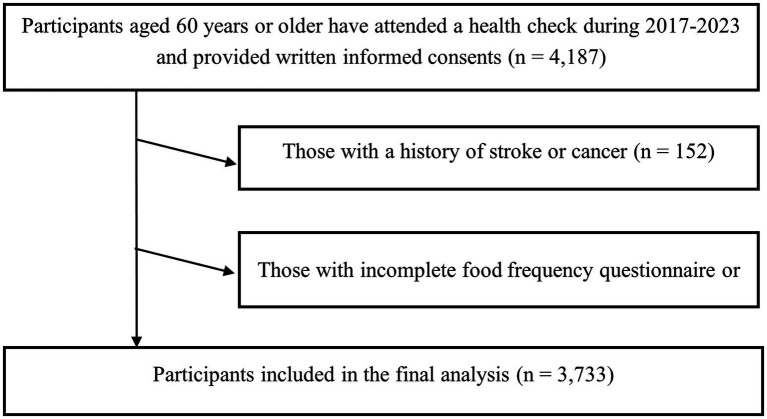
Study flow chart.

For the present analysis, data from 2017 to 2023 were used. Eligible participants were adults who underwent annual health examinations at health management and community centers in Tianjin, China, volunteered to take the structured questionnaire, and participated in standardized sarcopenia-related assessments. Data were cross-checked for internal consistency by trained staff, and any implausible or contradictory values were verified against original records and corrected when possible; otherwise, the affected participant’s data were treated as missing and excluded from the final analysis. Inclusion criteria were: (1) age ≥60 years, and (2) provision of written informed consent. Exclusion criteria were: (1) incomplete food frequency questionnaire (FFQ) or missing sarcopenia-related measurements (*n* = 302), (2) a documented history of stroke or cancer (*n* = 152), because sarcopenia in these individuals is more likely to be secondary to underlying disease processes, such as neurological impairment or cancer-related cachexia (40, 41), rather than the primary age-related sarcopenia investigated in this study. These conditions may substantially affect muscle mass, strength, and function, potentially confounding the associations of interest. After applying these criteria, 3,733 participants were included in the final cross-sectional analysis (Figure 1).

### 2.2 Assessment of sarcopenia

Sarcopenia was diagnosed according to the Asian Working Group for Sarcopenia (AWGS) 2019 criteria, defined as a low ASM plus low HGS and/or low physical performance (1).

Body weight and ASM were measured with a direct segmental multi-frequency bioelectrical impedance analyzer (InBody 720; Biospace Co., Ltd., Seoul, Korea). Height was measured using standardized, calibrated stadiometers. The appendicular skeletal muscle mass index (ASMI) was calculated as ASM (kg)/height^2^ (m^2^) and classified as low if <7.0 kg/m^2^ for men and <5.7 kg/m^2^ for women (1).

HGS was measured with a calibrated dynamometer (EH101; CAMRY, Guangdong, China). Two trials were performed for each hand, and the maximum value was recorded. Low HGS was defined as HGS < 28 kg for men and <18 kg for women (1).

Gait speed was assessed over a 4-m course at habitual pace, with time recorded by trained staff. Low physical performance was defined as gait speed < 1.0 m/s for both sexes (1).

### 2.3 Assessment of dietary intake

Dietary information for the preceding month was collected using a validated, modified version of a FFQ comprising 100 food items, adapted from the original validated 81-item FFQ (42). Additional items were incorporated to capture region-specific foods consumed by the elderly population in Tianjin, while retaining all core items from the original instrument. Participants indicated their consumption frequency for each food item by selecting from 7 predetermined categories ranging from “almost never eat” to “≥2 times/day” for solid foods and 8 predetermined categories from “almost never drink” to “≥4 cups/day” for beverages. Standardized portion sizes were specified for each food item based on the FFQ validation study. Total energy intake was calculated by aggregating the energy contributions from all food items using data from the Chinese Food Composition Tables. The reliability and validity of the FFQ was evaluated in a representative subsample (n = 150) of the TCLSIH cohort by administering the FFQ twice approximately 3 months apart and comparing results with four-day dietary records. The Spearman’s rank correlation coefficients between the repeated FFQs were 0.68 for energy intake and 0.69 for watermelon consumption. Correlations between the FFQ and four-day dietary records were 0.49 for energy intake and 0.72 for watermelon consumption. Comprehensive details regarding the validation methodology and structure of the FFQ have been documented elsewhere (43).

Participants reported their watermelon consumption frequency using the following response options: “almost never,” “<1 time/week,” “1 time/week,” “2–3 times/week,” “4–6 times/week,” “1 time/day,” and “≥2 times/day.” Based on the frequency distribution of responses, watermelon consumption was stratified into three analytical categories: “almost never,” “≤1 time/week,” and “≥2–3 times/week.” In this cohort, the average weekly watermelon consumption was quantified as 300 g for males and 250 g for females.

To assess overall dietary quality, distinct dietary patterns were derived from the FFQ data (excluding watermelon) using factor analysis with principal component analysis. Based on eigenvalues ≥1.5, scree plot examination, and factor interpretability, we identified three predominant dietary patterns: the “Traditional Oriental” pattern, the “Fruits and Sweet” pattern, and the “Animal Food” pattern.

### 2.4 Assessment of other variables

Sociodemographic and health behavior data were obtained from two sources: (1) hospital electronic medical record (EMR) system for patients’ name, sex, and registration information; and (2) selected items from a standardized health-related questionnaire administered during patient admission. Only relevant sociodemographic items used for the analysis are reported in the present study. Due to institutional policy and participant privacy protection, the full questionnaire cannot be shared publicly; however, the complete wording of the variables analyzed is provided in Supplementary Table 1. The education level and monthly household income were collected through the questionnaire (“Your highest educational qualification: Primary school; junior high school; senior high school; vocational school; associate degree; bachelor’s degree; postgraduate degree; other (specify)” and “What is your total monthly household income? < RMB 3,000; RMB 3,000–5,000; RMB 5,000–10,000; > RMB 10,000″). Educational level was categorized as junior (≤9 years of compulsory education, equivalent to middle school completion or below) or senior (>9 years of compulsory education, equivalent to high school completion or above). For lifestyle and health-related habits, smoking status was grouped in three: smoker, ex-smoker or nonsmoker and drinking status was classified as every day, sometime, ex-drinker or nondrinker by self-reporting. Current smoker referred to participants who reported smoking at the time of the survey. Ex-smoker referred to participants who reported having previously smoked but were not smoking at the time of the survey. Nonsmoker referred to participants who reported never smoking. Everyday drinker referred to participants who reported drinking alcohol every day. Sometime drinker referred to participants who reported drinking alcohol occasionally but not every day. Ex-drinker referred to participants who reported having previously consumed alcohol but were abstinent at the time of the survey. Nondrinker referred to participants who reported never drinking alcohol. Physical activity (PA) was quantified using the validated short form of the International Physical Activity Questionnaire (IPAQ), which captures activity over a seven-day period (44). Body mass index (BMI) was calculated as weight in kilograms divided by height in meters squared (kg/m^2^). Participants were classified according to Chinese BMI guidelines as underweight (<18.5 kg/m^2^), normal weight (18.5–23.9 kg/m^2^), overweight (24.0–27.9 kg/m^2^), or obese (≥28.0 kg/m^2^) (45). Personal disease history, including diabetes, hypertension, hyperlipidemia, cardiovascular disease, stroke, and cancer, was documented through a combination of self-reports, personal health records, and annual health examination data. Depressive symptoms were evaluated using the Chinese version of the Zung Self-Rating Depression Scale (46).

Potential confounders were selected based on prior evidence, theoretical relevance to sarcopenia etiology, and their established impact on both muscle health and dietary intake. Age and sex were included as fundamental demographic determinants because sarcopenia is an age-related condition and muscle mass/function differ markedly between genders (1). Socioeconomic indicators (educational level, household income) and medical history (diabetes, hypertension, hyperlipidemia, cardiovascular disease) were identified as important confounders in previous epidemiological studies investigating lifestyle-sarcopenia relationships (47, 48). Lifestyle factors (smoking, drinking, PA) were included due to their documented influence on muscle metabolism and dietary patterns (47). Total energy and protein intake, consistently reported as a critical confounder in diet-disease associations, was also adjusted for Willett et al. (49). Depressive symptoms and dietary patterns were incorporated to account for psychological and nutritional confounding, and other lycopene-rich food intake was added to control for lycopene sources (50, 51).

### 2.5 Sample size

We performed *a priori* power analysis using PASS 15.0 software to determine the minimum sample size required to detect a meaningful association between watermelon consumption and sarcopenia prevalence via logistic regression. Assuming a two-sided *α* = 0.05, statistical power = 80%, baseline sarcopenia prevalence of 12% in the non-consumption group, an odds ratio (OR) of 0.70 for consumers vs. non-consumers, and an exposure prevalence of 30%, the required sample size was 3,421 participants. The final analytic sample comprised 3,733 participants, exceeding the minimum required for adequate power, indicating our study was sufficiently powered to detect the expected associations.

### 2.6 Statistical analysis

Statistical analyses were conducted using Statistical Analysis System 9.4 edition for Windows (SAS Institute Inc., Cary, NC, United States). The distribution of continuous variables was assessed for normality using the Kolmogorov–Smirnov test. As all continuous variables did not follow a normal distribution, these variables were expressed as medians with interquartile ranges (IQRs), while categorical variables were presented as percentages. Potential outliers in continuous variables were screened using the IQR method, where values lying more than 1.5 × IQR below the first quartile or above the third quartile were flagged and verified against original records. Data entry errors were corrected, and no valid data points were excluded. As all variables included had complete data, and analyses were performed on the full dataset.

Given the small proportion of participants in the highest consumption categories (“4–6 times/week,” “1 time/day,” and “≥2 times/day”), these were combined into a single “≥2–3 times/week” category to ensure sufficient sample size and statistical power for regression analyses. Therefore, the participants were categorized into three groups according to the frequency of watermelon consumption: “almost never,” “≤1 time/week,” and “≥2–3 times/week.” Besides, we performed sensitivity analyses using finer classification of high-frequency consumers. The association between watermelon consumption and sarcopenia was evaluated using multiple logistic regression models. Five models were constructed to assess this relationship: Model 1 was unadjusted (crude model); Model 2 was adjusted for age, sex, and BMI; Model 3 was further adjusted for smoking status, drinking status, PA, educational level, household income, individual history of diseases (diabetes, hypertension, hyperlipidemia, and cardiovascular disease), depressive symptoms, total energy intake, and dietary pattern; Model 4 was adjusted for variables in model 3 plus tomato intake; and Model 5 was adjusted for variables in model 3 plus lycopene-rich food and protein intake. ORs with 95% confidence intervals (CIs) were calculated, with the “almost never” group serving as the reference category. *P*-values for trend were determined to assess dose–response relationships.

To investigate potential effect modification, stratified analyses were performed by major covariates including sex, BMI categories (<18.5, 18.5–<24, 24–<28, and ≥28 kg/m^2^), PA (<23.0 and ≥23.0 metabolic equivalent (MET)-h/week), smoking status (current smoker, nonsmoker, and ex-smoker), drinking status (everyday, sometime, ex-drinker, and nondrinker), education level (≥senior and <senior), household income (≥3,000 and <3,000 yuan/month), presence of chronic diseases (diabetes, hypertension, hyperlipidemia, and cardiovascular disease), and depressive symptoms score (>45.0 and ≤45.0). The likelihood ratio test was used to calculate *p*-values for interaction.

All statistical tests were two-sided, and *p*-values <0.05 were considered statistically significant.

## 3 Results

### 3.1 Characteristics of participants

In the present study, the prevalence of sarcopenia was 12.6% (471/3,733). Of the 3,733 participants included in the analysis, 43.7% (n = 1,630) were men. The median age was 65.8 years (IQR: 62.1–70.0 years), and the median BMI was 25.0 kg/m^2^ (IQR: 22.7–27.3 kg/m^2^). Regarding smoking status, 33.3% were current smokers, 61.3% were nonsmokers, and 5.46% were ex-smokers. PA levels showed a median of 15.8 MET-h/week. Chronic disease prevalence was substantial, with 60.4% having hypertension, 60.5% having hyperlipidemia, 22.6% having diabetes, and 8.33% having cardiovascular disease. Detailed participant characteristics are comprehensively presented in Table 1.

**Table 1:** Participant characteristics (*n* = 3,733)^a^.

Characteristics	All	Men (43.7%)	Women (56.3%)
No. of subjects	3,733	1,630	2,103
Age (years)	65.8 (62.1, 70.0)	66.0 (63.0, 71.0)	65.0 (62.0, 69.0)
BMI (kg/m^2^)	25.0 (22.7, 27.3)	24.6 (22.5, 26.9)	25.3 (23.0, 27.6)
PA (≥23.0 MET-h/wk.)	15.8 (8.68, 28.2)	15.8 (7.42, 37.7)	15.8 (9.94, 24.7)
Total energy intake (kcal/day)	1862.3 (1483.3, 2323.8)	2110.9 (1708.2, 2558.7)	1687.6 (1385.0, 2063.4)
“Traditional Oriental” dietary pattern score	−0.15 (−0.67, 0.49)	0.02 (−0.57, 0.75)	−0.24 (−0.75, 0.32)
“Fruits and sweets” dietary pattern score	−0.21 (−0.62, 0.35)	−0.19 (−0.68, 0.41)	−0.22 (−0.58, 0.31)
“Animal foods” dietary pattern score	−0.21 (−0.66, 0.39)	0.16 (−0.42, 0.88)	−0.40 (−0.73, 0.02)
Smoking status (%)			
Current smoker	33.3	41.5	26.9
Nonsmoker	61.3	50.4	69.7
Ex-smoker	5.46	8.10	3.43
Drinker status (%)			
Everyday	14.1	30.1	1.84
Sometime	6.78	12.9	2.08
Ex-drinker	1.86	4.15	0.10
Nondrinker	77.2	52.9	96.0
Educational level (≥senior grade, %)	5.32	9.56	1.89
Household income (≥3,000 Yuan/m, %)	11.1	12.0	10.4
Individual history of diseases (%)			
Diabetes	22.6	19.0	25.3
Hypertension	60.4	62.9	58.5
Hyperlipidemia	60.5	47.2	70.7
Cardiovascular disease	8.33	5.71	10.4
Depressive symptoms score (>45, %)	8.15	6.01	9.83

BMI, body mass index; PA, physical activity; MET, metabolic equivalent. ^a^Values are medians [IQRs] for continuous variables and percentages for categorical variables.

### 3.2 Relationship between watermelon consumption and sarcopenia risk

Table 2 presents the crude and adjusted relationships between watermelon consumption frequency and sarcopenia risk. In the crude model (Model 1), compared to participants who almost never consumed watermelon (reference group), those who consumed watermelon ≤1 time/week had a 28% lower odds of sarcopenia (OR = 0.72; 95% CI: 0.56–0.92), while those who consumed watermelon ≥2–3 times/week had a 52% lower odds (OR = 0.48; 95% CI: 0.30–0.73). The trend test showed a significant inverse association between watermelon consumption frequency and sarcopenia risk (*p* for trend <0.0001). After adjusting for age, sex, and BMI (Model 2), the inverse association remained significant, with ORs of 0.66 (95% CI, 0.51–0.86) for the ≤1 time/week group and 0.48 (95% CI, 0.29–0.74) for the ≥2–3 times/week group (*p* for trend <0.0001). Further adjustment for smoking status, drinking status, PA, educational level, household income, individual history of diseases (diabetes, hypertension, hyperlipidemia, and cardiovascular disease), depressive symptoms, total energy intake, and dietary pattern (Model 3) slightly attenuated the association, but it remained significant with ORs of 0.74 (95% CI, 0.56–0.98) for the ≤1 time/week group and 0.52 (95% CI, 0.31–0.83) for the ≥2–3 times/week group (*p* for trend <0.01). In Model 4, which controlled for the variables in Model 3 plus tomato intake, the associations remained consistent with ORs of 0.74 (95% CI, 0.55–0.97) for the ≤1 time/week group and 0.51 (95% CI, 0.31–0.82) for the ≥2–3 times/week group (*p* for trend <0.01). Similarly, in Model 5, which controlled for the variables in Model 3 plus lycopene-rich food intake and protein intake, comparable results were observed: ORs of 0.72 (95% CI, 0.54–0.95) and 0.49 (95% CI, 0.29–0.79) for the respective groups (*p* for trend <0.001). We examined watermelon consumption using a finer classification. The protective association remained consistent in direction across all consumption levels (Supplementary Table 2).

**Table 2:** Adjusted relationships of frequency of watermelon consumption to sarcopenia (*n* = 3,733).

Logistic regression models	Frequency of watermelon consumption			*p* for trend^a^
	Almost never	≤1 time/week	≥2–3 time/week	
No. of subjects	2,621	806	306	-
No. of sarcopenia	365	84	22	-
Model 1^b^	1.00 (Ref)	**0.72 (0.56, 0.92)** ^**c**^	**0.48 (0.30, 0.73)**	**<0.0001**
Model 2^d^	1.00 (Ref)	**0.66 (0.51, 0.86)**	**0.48 (0.29, 0.74)**	**<0.0001**
Model 3^e^	1.00 (Ref)	**0.74 (0.56, 0.98)**	**0.52 (0.31, 0.83)**	**<0.01**
Model 4^f^	1.00 (Ref)	**0.74 (0.55, 0.97)**	**0.51 (0.31, 0.82)**	**<0.01**
Model 5^g^	1.00 (Ref)	**0.72 (0.54, 0.95)**	**0.49 (0.29, 0.79)**	**<0.001**

Significant values (*p* < 0.05) are shown in bold. ^a^Analysis by multiple logistic regression model. ^b^Model 1 was crude model. ^c^Odds ratio (95% confidence interval) (all such values). ^d^Model 2 was adjusted for age, sex, and BMI. ^e^Model 3 was adjusted for variables in model 2 plus smoking status, drinking status, physical activity, educational level, household income, individual history of diseases (diabetes, hypertension, hyperlipidemia and cardiovascular disease), depressive symptoms, total energy intake, and dietary pattern. ^f^Model 4 was adjusted for variables in model 3 plus tomato intake. ^g^Model 5 was adjusted for variables in model 3 plus lycopene-rich food (except watermelon) and protein intake.

### 3.3 Stratified analyses

To further explore the relationship between watermelon consumption and sarcopenia risk across different population subgroups, we conducted stratified analyses by major covariates (Table 3). Stratified analyses revealed notable variations across different population subgroups. Among men, watermelon consumption ≥2–3 times/week was associated with significantly lower odds of sarcopenia (OR = 0.34; 95% CI: 0.15–0.69; *p* for trend <0.01), while this association was not significant in women (OR = 0.76; 95% CI: 0.37–1.46; *p* for trend = 0.28). The protective effect was more pronounced in participants with BMI 24–28 kg/m^2^ (OR = 0.30; 95% CI: 0.07–0.91; *p* for trend = 0.03). Among nonsmokers, higher watermelon consumption showed strong protective effects (OR = 0.45; 95% CI: 0.22–0.87; *p* for trend <0.01), whereas the association was not significant in current smokers. Notably, participants without hyperlipidemia demonstrated stronger protective associations (OR = 0.38; 95% CI: 0.16–0.83; *p* for trend <0.01) compared to those with hyperlipidemia (OR = 0.66; 95% CI: 0.34–1.21; *p* for trend = 0.25), with a significant interaction (*p* for interaction = 0.02).

**Table 3:** Relationships between frequency of watermelon consumption and risk of sarcopenia stratified by major covariates.

Major covariates	*n*	Frequency of watermelon consumption			*p* for trend^a^	*p* for interaction^b^
		Almost never	≤1 time/week	≥2–3 time/week		
Sex						0.17
Men	1,630	1.00 (reference)	0.67 (0.43, 1.00) ^c^	**0.34 (0.15, 0.69)**	**<0.01**	
Women	2,103	1.00 (reference)	0.83 (0.55, 1.24)	0.76 (0.37, 1.46)	0.28	
BMI, kg/m^2^						0.64
0 ≤ BMI < 18.5	77	1.00 (reference)	1.13 (0.11, 12.6)	-	0.41	
18.5 ≤ BMI < 24	1,375	1.00 (reference)	0.82 (0.57, 1.16)	0.60 (0.33, 1.04)	0.055	
24 ≤ BMI < 28	1,539	1.00 (reference)	0.66 (0.35, 1.18)	**0.30 (0.07, 0.91)**	**0.03**	
BMI ≥ 28	742	1.00 (reference)	0.58 (0.09, 2.54)	0.46 (0.02, 3.97)	0.42	
PA, MET-h/wk						0.36
≥23.0	2,527	1.00 (reference)	0.81 (0.46, 1.39)	0.39 (0.12, 1.00)	0.07	
<23.0	1,206	1.00 (reference)	**0.69 (0.49, 0.97)**	**0.54 (0.30, 0.93)**	**<0.01**	
Smoking status						0.42
Current smoker	1,241	1.00 (reference)	0.70 (0.42, 1.12)	0.87 (0.37, 1.85)	0.28	
Nonsmoker	2,288	1.00 (reference)	0.69 (0.46, 1.01)	**0.45 (0.22, 0.87)**	**<0.01**	
Ex-smoker	204	1.00 (reference)	0.97 (0.12, 7.18)	-	0.56	
Drinking status						0.81
Everyday	528	1.00 (reference)	0.45 (0.17, 1.11)	0.80 (0.15, 3.24)	0.22	
Sometime	253	1.00 (reference)	1.48 (0.19, 9.28)	34.9 (1.54, 949.5)	0.07	
Ex-drinker	69	1.00 (reference)	-	-	0.48	
Nondrinker	2,883	1.00 (reference)	0.83 (0.60, 1.14)	**0.43 (0.22, 0.76)**	**<0.01**	
Education level						0.86
≥Senior	199	1.00 (reference)	-	-	0.96	
<Senior	3,534	1.00 (reference)	**0.75 (0.56, 0.99)**	**0.50 (0.30, 0.81)**	**<0.01**	
Household income, yuan/m						0.58
≥3,000	415	1.00 (reference)	2.20 (0.29, 18.7)	1.01 (0.02, 20.8)	0.68	
<3,000	3,318	1.00 (reference)	**0.72 (0.54, 0.97)**	**0.48 (0.28, 0.78)**	**<0.01**	
Diabetes						0.65
Yes	845	1.00 (reference)	1.16 (0.41, 3.05)	0.45 (0.06, 2.58)	0.65	
No	2,888	1.00 (reference)	**0.71 (0.52, 0.96)**	**0.48 (0.28, 0.80)**	**<0.01**	
Hypertension						0.92
Yes	2,255	1.00 (reference)	0.81 (0.54, 1.20)	**0.41 (0.18, 0.86)**	**0.02**	
No	1,478	1.00 (reference)	0.73 (0.47, 1.09)	0.58 (0.29, 1.08)	**0.04**	
Hyperlipidemia						**0.02**
Yes	2,258	1.00 (reference)	0.94 (0.64, 1.36)	0.66 (0.34, 1.21)	0.25	
No	1,475	1.00 (reference)	**0.51 (0.32, 0.81)**	**0.38 (0.16, 0.83)**	**<0.01**	
Cardiovascular disease						0.24
Yes	311	1.00 (reference)	0.31 (0.08, 1.05)	0.16 (0.01, 1.38)	**0.03**	
No	3,422	1.00 (reference)	0.78 (0.58, 1.04)	**0.55 (0.32, 0.89)**	**<0.01**	
Depressive symptoms score						0.81
>45.0	304	1.00 (reference)	0.48 (0.13, 1.52)	1.24 (0.10, 10.6)	0.47	
≤45.0	3,429	1.00 (reference)	0.75 (0.56, 1.01)	**0.50 (0.29, 0.81)**	**<0.01**	

Significant values (*p* < 0.05) are shown in bold. BMI, body mass index; PA, physical activity; MET, metabolic equivalent. ^a^Analysis by multiple logistic regression model. Adjusted for age, sex, BMI, smoking status, drinking status, PA, educational level, household income, individual history of diseases (diabetes, hypertension, hyperlipidemia and cardiovascular disease), depressive symptoms, total energy intake and dietary patterns. ^b^*P* for interaction was calculated using the likelihood ratio test. ^c^ORs (95% CIs) (all such values).

## 4 Discussion

Our study demonstrates a significant inverse association between watermelon consumption and sarcopenia prevalence in an elderly Chinese population from the TCLSIH cohort. After adjusting for multiple confounding factors, participants with higher watermelon consumption showed substantially lower odds of sarcopenia compared to those with lower consumption. While the association was generally consistent across most population subgroups, individuals with hyperlipidemia showed no significant protective benefit, suggesting that lipid metabolism status might modify watermelon’s beneficial effects on sarcopenia risk.

In this cohort, the average weekly watermelon consumption was quantified as 300 g for males and 250 g for females. Participants consuming watermelon “≤1 time/week” had an estimated weekly intake of approximately 250–300 grams, while those consuming “≥2–3 times/week” had an estimated weekly intake of 500–900 grams. According to the fully adjusted analysis, even modest watermelon consumption (250–300 g/week) was associated with 28% lower odds of sarcopenia, while moderate consumption (500–900 g/week) was associated with 51% lower odds. This dose–response relationship suggests that the L-citrulline content in these consumption amounts, approximately 0.4–0.7 grams for low consumption and 0.8–3.15 grams for moderate consumption based on watermelon’s L-citrulline content of 1.6–3.5 grams per kilogram, might be sufficient to exert meaningful biological effects on muscle health (23, 52).

While no previous studies have specifically examined watermelon consumption in relation to sarcopenia, several investigations have explored the relationship between fruit consumption or specific nutrients abundant in watermelon and muscle health outcomes. A recent meta-analysis reported that antioxidant-rich fruits and vegetables are linked to a 30–40% risk reduction (22), which is comparable in magnitude to our estimates for higher watermelon consumption. Figueroa et al. (29) demonstrated that L-citrulline supplementation (10 g/day) combined with resistance training significantly improved leg lean mass and muscle strength in hypertensive postmenopausal women. Similarly, Wen et al. showed that lycopene supplementation increased the proportion of slow-twitch muscle fibers through AMPK signaling pathway activation, enhancing muscle anti-fatigue capacity in animal models (33). Prospective data from the Framingham Offspring Study showed that higher total carotenoid intake was associated with reduced decline in HGS and gait speed, phenotypes integral to sarcopenia definitions and consistent with our findings (27). However, our study provides important complementary evidence by examining whole food consumption rather than isolated nutrient supplementation. Watermelon provides L-citrulline alongside other potentially beneficial nutrients including lycopene, *β*-carotene, and vitamin C (23, 24, 53), which may work synergistically to support muscle health through multiple pathways including enhanced NO synthesis, reduced oxidative stress, and improved protein synthesis. This whole-food approach may be more practical and sustainable for population-level sarcopenia prevention compared to isolated supplement interventions.

Several potential biological mechanisms may explain the inverse association between watermelon consumption and sarcopenia observed in our study. First, L-citrulline functions as a precursor to L-arginine, which is subsequently converted to NO (54, 55). L-citrulline supplementation represents a more efficient method for elevating plasma L-arginine levels compared to direct L-arginine supplementation (56). This enhanced conversion improves NO bioavailability in muscle tissues (56). NO acts as a vasodilator through cyclic guanosine monophosphate (cGMP)-mediated phosphorylation (57). This vasodilatory action improves nutrient and oxygen delivery to active muscle tissues (58). Enhanced delivery supports adenosine triphosphate (ATP) and phosphocreatine resynthesis during physical exertion (58). These physiological effects may help sustain muscle force production during repeated contractions. Beyond enhancing circulatory dynamics, NO may improve muscle function through additional mechanisms, including facilitation of ATP hydrolysis and reduction in the metabolic cost of force production (59).

Furthermore, NO bioavailability might be augmented through a nitric oxide synthase (NOS)-dependent L-arginine-NO pathway, with L-citrulline and dietary nitrates serving as critical precursors (60). Recent findings by Sureda et al. (61) have demonstrated that L-citrulline supplementation increases nitrogen availability, which consequently supports protein synthesis in muscle tissue. Complementary evidence from animal models strengthens these observations; notably, in aged rats, a one-week diet enriched with L-citrulline (5 g/kg per day) significantly enhanced absolute muscle protein synthesis rates and increased protein mass (62). Therefore, L-citrulline, as a precursor to NO, may improve muscle function through multiple pathways: enhancing nutrient delivery, reducing ATP utilization costs, and promoting muscle protein synthesis. Further investigations are warranted to elucidate the precise mechanisms underlying the relationship between watermelon consumption and sarcopenia prevention in elderly populations.

Our stratified analyses demonstrated that watermelon’s protective association against sarcopenia remained consistent across most subgroups, except among individuals with hyperlipidemia, suggesting dyslipidemia may nullify watermelon’s protective effects. This finding might be explained by dyslipidemia’s overwhelming catabolic metabolic environment. Dyslipidemia represents a systemic metabolic disorder characterized by heightened oxidative stress, chronic inflammation, and endothelial dysfunction (63). Oxidized low density lipoprotein triggers inflammatory cascades and reactive oxygen species production, creating a pro-inflammatory state that independently promotes sarcopenia pathogenesis (64). In normolipidemic individuals, baseline inflammation and oxidative stress levels are relatively low, allowing watermelon’s antioxidant, anti-inflammatory, and vasodilatory benefits to meaningfully counteract age-related muscle decline. However, in dyslipidemic individuals, pathologically elevated systemic inflammation, oxidative stress, and endothelial dysfunction create a different scenario (63, 65). The endothelial dysfunction characteristic of dyslipidemia directly impairs NO bioavailability, counteracting the mechanism by which watermelon’s L-citrulline provides benefits (65). The magnitude of pro-catabolic stimuli from dyslipidemia likely overwhelms watermelon’s modest protective effects, diminishing its impact to non-significance against this pathological backdrop. Furthermore, statins, common lipid-lowering medications, may cause muscle damage and muscle atrophy through mechanisms involving effects on mitochondrial function, muscle energy metabolism, and protein degradation (66–68). These findings suggest that lipid metabolism status might modify watermelon’s beneficial effects, though underlying mechanisms require further investigation.

In addition to the biological plausibility, the observed association carries clinical and public health relevance. A 28–51% reduction in the odds of sarcopenia is comparable to many established lifestyle interventions, such as consumption of protein, fruit and vegetable or PA, which often yield about 40% risk reductions (22, 69, 70). Given the prevalence of sarcopenia in 14–20% of Chinese older adults (7–10), even modest relative reductions might translate into substantial population-level benefits. In our study population with a 12.6% prevalence, regular watermelon consumption (≥2–3 times/week) might theoretically prevent approximately 6–7 cases per 100 elderly individuals, underscoring its potential as a simple, accessible, and affordable dietary strategy for sarcopenia prevention. Besides, our findings suggested that the effectiveness of watermelon consumption may be context-dependent and influenced by underlying metabolic conditions. Healthcare providers should consider patients’ lipid profiles when recommending dietary strategies for sarcopenia prevention, as individuals with dyslipidemia may require more intensive interventions or adjunctive lipid management to realize the benefits of functional foods.

The main strength of our study is the large sample size, extensive information on other lifestyle factors, and use of a validated FFQ for assessment of dietary consumption. Additionally, when we adjusted for a multitude of potential confounders the results did not change materially, indicating the robustness of our findings. What’s more, the definition of sarcopenia in this study is strictly based on the criterion of AWGS2019, which is more adaptable to the sample population in our study, and may reduce the bias of misclassification to a certain extent. This study also has several limitations. First, self-reported watermelon information is subject to measurement errors. In view of this situation, unreliable FFQs were excluded in our study. Second, muscle mass was measured by bioelectrical impedance analysis rather than the gold standard test dual-energy X-ray absorptiometry. However, results of muscle mass estimation using bioelectrical impedance analysis are highly correlated with that measured using dual-energy X-ray absorptiometry (71). Third, as with any epidemiological study, we cannot completely rule out the possibility of residual or unmeasured confounding. Several important factors were not captured in our analysis, including consumption of other bioactive-rich foods, use of medications that may affect muscle metabolism (including statins, corticosteroids, or angiotensin-converting enzyme inhibitors), and indicators of physical frailty or functional status beyond our measured parameters. Fourth, the cross-sectional design inherently limits causal inference, as we cannot establish temporal relationships between watermelon consumption and sarcopenia development. It remains possible that individuals with better muscle health are more likely to maintain healthier dietary patterns, including higher fruit consumption, creating potential reverse causation bias (72). Fifth, while PA was quantified using validated METs from the IPAQ, our assessment lacked specificity regarding the type of exercise performed. Resistance training and aerobic exercise have differential impacts on muscle mass preservation, with resistance exercise being particularly beneficial for maintaining and building skeletal muscle mass (73). The inability to distinguish between these exercise modalities may have introduced residual confounding, as individuals with higher watermelon consumption might also engage in more beneficial exercise patterns for muscle health. Last, generalizing our results to other populations requires caution due to variations in dietary patterns, genetic polymorphisms, baseline nutritional status, and chronic disease management across populations. Dietary habits and food preparation methods vary substantially across cultures. Western populations typically consume different fruit varieties, portion sizes, and processing methods compared to Chinese populations, which might affect the bioavailability of L-citrulline and other bioactive compounds found in watermelon (74). Genetic polymorphisms affecting NO metabolism, particularly variants in NOS genes, show significant ethnic variation and may influence individual responses to L-citrulline intake (75). Baseline nutritional status varies across different populations, which may modulate the relationship between watermelon consumption and muscle health outcomes (76). Additionally, while our primary analysis showed consistent associations, the limited significant interactions in stratified analyses suggest that the relationship may vary across certain population subgroups, which warrants careful consideration when generalizing findings.

In conclusion, our study provides novel evidence of an inverse association between watermelon consumption and sarcopenia prevalence in an elderly Chinese population. The rich nutrient profile of watermelon, including L-citrulline, offers biologically plausible mechanisms for this relationship. Future research should include well-designed RCTs to verify causality, such as L-citrulline supplementation or controlled watermelon intake in elderly individuals with sarcopenia or pre-sarcopenia. Dose–response RCTs comparing varying watermelon consumption levels could identify optimal intake. Long-term prospective cohorts in different ethnic and geographic populations would clarify temporal relationships and generalizability. Mechanistic studies examining changes in muscle protein synthesis, NO metabolism, and inflammation biomarkers will help elucidate underlying pathways. If confirmed, increasing watermelon consumption might represent a simple, accessible dietary strategy to help combat the growing public health challenge of sarcopenia in aging populations worldwide.

## Data Availability

The raw data supporting the conclusions of this article will be made available by the authors, without undue reservation.

## Glossary

AMPK: Adenosine 5′-monophosphate activated protein kinase
ASM: Appendicular skeletal muscle mass
ASMI: Appendicular skeletal muscle mass index
ATP: Adenosine triphosphate
AWGS: Asian Working Group for Sarcopenia
BMI: Body mass index
cGMP: Cyclic guanosine monophosphate
CI: Confidence interval
CRP: C-reactive protein
EMR: Electronic medical record
EWGSOP: European Working Group on Sarcopenia in Older People
FFQ: Food frequency questionnaire
HGS: Handgrip strength
IL-6: Interleukin-6
IPAQ: International physical activity questionnaire
IQR: Interquartile range
MET: Metabolic equivalent
NO: Nitric oxide
NOS: Nitric oxide synthase
OR: Odds ratio
PA: Physical activity
PGC-1α: Peroxisome proliferator-activated receptor-*γ* coactivator-1α
RCT: Randomized controlled trial
TCLSIH: Tianjin chronic low-grade systemic inflammation and health
