# Supplementary material for: The relationship between watermelon consumption and sarcopenia in an elderly general population: findings from the Tianjin chronic low-grade systemic inflammation and health study
Source: Front Nutr. 2025 Sep 30;12:1663996. doi: 10.3389/fnut.2025.1663996 (PMC12518088; doi:10.3389/fnut.2025.1663996)
Supplement: Supplementary file 1 [file Table_1.DOCX]

| **Supplementary Table 1**. Sociodemographic and health behavior items and response options used in the present study | | |
| --- | --- | --- |
| Variable | Question wording (Chinese/English translation) | Response options (Chinese/English translation) |
| Smoking status | 您的吸烟习惯/Your smoking habits | □吸烟/Smoke (currently) □从来没吸过/Never smoked □以前吸烟，现在戒了/Used to smoke, now quit |
| Drinker status | 您现在喝酒吗?/Do You Drink Alcohol Now? | □每天喝/Drink every day □偶尔喝/Drink occasionally □以前喝过，现在不喝了/Used to drink, but don’t now □从来没喝过/Have never drunk |
| Education level | 您的最高学历/Your Highest Educational Qualification | □小学毕业/Primary school □初中毕业/Junior high school □高中毕业/Senior high school □中专毕业/Vocational school □大专毕业/Associate degree □本科毕业/Bachelor’s degree □研究生毕业/Postgraduate degree □其它 ( )/Other (specify) |
| Monthly household income | 您每月家庭总收入是多少?/What is your total monthly household income? | □3000 元以下/< RMB 3,000 □3000-5000 元/RMB 3,000–5,000 □5000-10,000 元/RMB 5,000–10,000 □10,000 元以上/> RMB 10,000 |

| **Supplementary Table 2.** Adjusted relationships of frequency of watermelon consumption to sarcopenia (n = 3,733). | | | | | | | |
| --- | --- | --- | --- | --- | --- | --- | --- |
|  | Frequency of watermelon consumption | | | | |  | *p* for trend ^a^ |
|  | Almost never | ≤1 time/week | 2-3 time/week | 4-6 time/week | 1 time/day | ≥2 times/day |  |
| No. of subjects | 2,621 | 806 | 237 | 49 | 20 | 0 | - |
| No. of sarcopenia | 365 | 84 | 15 | 5 | 2 | 0 | - |
| Model 1 ^b^ | 1.00 (Ref) | **0.72 (0.56, 0.92)** ^c^ | **0.42 (0.24, 0.69)** | 0.70 (0.24, 1.62) | 0.69 (0.11, 2.39) | - | **<0.001** |
| Model 2 ^d^ | 1.00 (Ref) | **0.66 (0.51, 0.86)** | **0.42 (0.23, 0.71)** | 0.63 (0.21, 1.53) | 0.72 (0.11, 2.75) | - | **<0.001** |
| Model 3 ^e^ | 1.00 (Ref) | **0.75 (0.56, 0.99)** | **0.46 (0.25, 0.80)** | 0.68 (0.22, 1.74) | 0.79 (0.12, 3.15) | - | **<0.01** |
| Model 4 ^f^ | 1.00 (Ref) | **0.74 (0.55, 0.97)** | **0.46 (0.25, 0.79)** | 0.68 (0.22, 1.73) | 0.73 (0.11, 2.93) | - | **<0.01** |
| Model 5 ^g^ | 1.00 (Ref) | **0.72 (0.54, 0.95)** | **0.43 (0.23, 0.74)** | 0.74 (0.24, 1.88) | 0.68 (0.10, 2.70) |  | **<0.01** |
| Significant values (*p* < 0.05) are shown in bold. | | | | | | | |
| ^a^ Analysis by multiple logistic regression model. | | | | | | | |
| ^b^ Model 1 was crude model. | | | | | | | |
| ^c^ Odds ratio (95% confidence interval) (all such values). | | | | | | | |
| ^d^ Model 2 was adjusted for age, sex, and BMI. | | | | | | | |
| ^e^ Model 3 was adjusted for variables in model 2 plus smoking status, drinking status, physical activity, educational level, household income, individual history of diseases (diabetes, hypertension, hyperlipidemia and cardiovascular disease), depressive symptoms, total energy intake, and dietary pattern. | | | | | | | |
| ^f^ Model 4 was adjusted for variables in model 3 plus tomato intake. | | | | | | | |
| ^g^ Model 5 was adjusted for variables in model 3 plus lycopene-rich food (except watermelon) and protein intake. | | | | | | | |
